# Effectiveness of low-level laser therapy compared to standard postoperative care on pain intensity and cesarean section wound healing in the immediate postoperative period: a randomized clinical trial

**DOI:** 10.1007/s10103-026-04934-0

**Published:** 2026-06-25

**Authors:** Maria Luci Quirino de Melo Trindade, Alexandre Delgado, Yuri Miranda de Alencar, Yasmin Eduarda da Silva, Maria Eduarda da Conceição Silveira, Andrea Lemos

**Affiliations:** 1 Federal University of Pernambuco, Recife, Brazil; 2 Instituto de Medicina Integral Professor Fernando Figueira, Recife, Brazil

**Keywords:** Low-level laser therapy, Cesarean section, Analgesia, Phototherapy

## Abstract

This study aimed to evaluate the effectiveness of low-level laser therapy (LLLT) in reducing pain intensity and improving wound healing quality in women during the immediate postoperative period following cesarean section. This randomized clinical trial included 104 postpartum women recruited between April and October 2024, who were allocated into two groups: a control group (*n* = 52) and an experimental group (*n* = 52). The experimental group received infrared LLLT at a dose of 5 J/cm². Pain intensity was assessed using the Visual Analog Scale (VAS), and wound healing was evaluated using the Vancouver Scar Scale (VSS). Two LLLT sessions were performed at 12–24 h and 48 h postpartum. A significant reduction in pain intensity was observed after the second application (mean difference [MD]: −1.4; 95% CI: −2.3 to − 0.5), as well as an improvement in overall wound healing quality (MD: 3.31; 95% CI: 3.19 to 3.43). Domain-specific analysis of the VSS revealed improvements in vascularity (MD: 1.0; 95% CI: 1.00 to 1.03) and scar pliability (MD: 1.11; 95% CI: 1.07 to 1.15). No significant differences were observed in global health perception or in the need for pharmacological analgesia. These findings suggest that LLLT is effective in reducing postoperative pain intensity and improving wound healing quality—particularly in terms of scar vascularity and pliability—in women undergoing cesarean section.

Clinical trial registration

This study was prospectively registered in the Brazilian Registry of Clinical Trials (ReBEC) under the registration number RBR-4pkhf4b.

## Introduction

With the gradual increase in cesarean section rates, particularly in developing countries, there has been a growing need to investigate and develop strategies to prevent adverse outcomes associated with this procedure, aiming to reduce postoperative pain intensity and discomfort at the surgical wound site [1]. Cesarean delivery is associated with several postoperative repercussions, including mood disturbances, prolonged recovery time, reduced quality of life, delayed return to activities of daily living, impaired bonding with the newborn, partners, and family members, neglect of self-care, as well as impaired wound healing and musculoskeletal pain near or at the site of the surgical incision [2].

Women undergoing cesarean section are at increased risk of developing persistent pain at the surgical scar site [3]. Pharmacological strategies commonly used to alleviate these symptoms in the immediate postpartum period include scheduled administration of nonsteroidal anti-inflammatory drugs and opioids. However, there is increasing interest in optimizing non-pharmacological approaches, such as the use of low-level laser therapy (LLLT).

LLLT has been applied for more than five decades and is widely used across diverse clinical settings [4]. It is a non-invasive and safe therapeutic modality that, through photobiomodulation, delivers photons at non-thermal irradiance levels to stimulate biological responses involved in tissue repair [5]. Upon reaching the tissue, light interacts with photosensitive cells known as endogenous chromophores, including water, melanin, and blood components [5]. LLLT has been proposed as an adjunctive treatment for wound healing, particularly in surgical wounds, with the aim of preventing potential complications such as persistent pain and impaired healing processes [6].

Given the growing interest in reducing pain intensity and improving scar quality in the postoperative period following cesarean section, LLLT has been increasingly investigated and applied in clinical practice. Postoperative wound pain after cesarean delivery is often intense and is associated with a 2.5-fold higher risk of progression to persistent pain and a threefold increased risk of postpartum depression [7].

In light of these considerations, the present study aimed to evaluate the effectiveness of low-level laser therapy compared with standard postoperative care in reducing pain intensity and improving wound healing following cesarean section during the immediate postoperative period.

## Methods

This study was conducted as a randomized controlled clinical trial comparing low-level laser therapy (LLLT) with standard postoperative care in women who underwent cesarean section, in accordance with the Consolidated Standards of Reporting Trials (CONSORT) guidelines. The trial was registered in the Brazilian Registry of Clinical Trials (REBEC) under the identification number RBR-4pkhf4b. The study was carried out at the Obstetric Center of the Hospital das Clínicas of the Federal University of Pernambuco (HC-UFPE), located in Recife, Pernambuco, Brazil, between May and October 2024. Data collection commenced after approval by the Institutional Research Ethics Committee (approval number: 6.702.016; CAAE: 77075924.4.0000.5208), granted on March 25, 2024. All participants provided written informed consent prior to enrollment.

The study setting was a public university hospital that serves as a tertiary referral center for high-complexity obstetric care and predominantly assists women covered by the Brazilian Unified Health System (SUS). The institution receives patients from both the Recife Metropolitan Area and rural regions of the state of Pernambuco, resulting in a population with diverse sociodemographic and clinical profiles. The participants were postpartum women hospitalized in the immediate postoperative period following cesarean section, reflecting routine clinical practice within the hospital setting.

Randomization to receive LLLT in addition to standard care or standard care alone was performed using a computer-generated random number sequence prepared by an individual not involved in the study, using Random Allocation Software version 1.0 (Isfahan, Iran, 2004). Allocation concealment was ensured through the use of sequentially numbered, sealed, opaque black envelopes, each corresponding to group assignment according to the randomization list.

Sample size calculation was based on data from a previous randomized clinical trial (de Holanda Araújo et al., 2019), which identified a difference in mean pain intensity between the control group (mean = 5.23; SD = 2.41) and the intervention group (mean = 3.90; SD = 2.40). Assuming a statistical power of 80% and a two-sided significance level of 5% (α = 0.05), the estimated sample size was 104 participants, equally allocated between the two groups (52 per group), as calculated using OpenEpi software (version 3.0).

The study population consisted of postpartum women admitted to the obstetric center during the immediate postoperative period following cesarean section. Eligibility criteria included: (1) age ≥ 18 years; (2) postoperative period between 24 and 30 h; and (3) a mean pain score of at least 3 on the Visual Analog Scale (VAS) for surgical wound pain. Exclusion criteria were: (1) impaired communication during the postpartum period; and (2) presence of clinical complications, such as hemorrhage, wound dehiscence, or sepsis.

The intervention was administered by physiotherapists and physiotherapy students who were previously trained in laser application procedures. A diode laser device (Laser Power Therapy, ECCO^®^ Reability) with an output power of 120 mW was used. The equipment was previously calibrated and evaluated by an independent professional to ensure accurate delivery of the prescribed dose.

In the experimental group, LLLT was applied in continuous mode using infrared light at a dose of 5 J/cm², power output of 120 mW and wavelength of 660 nm. The application time was 42 s per point, with points demarcated at 1-cm intervals along the entire length of the surgical incision.

**Fig. 1:**
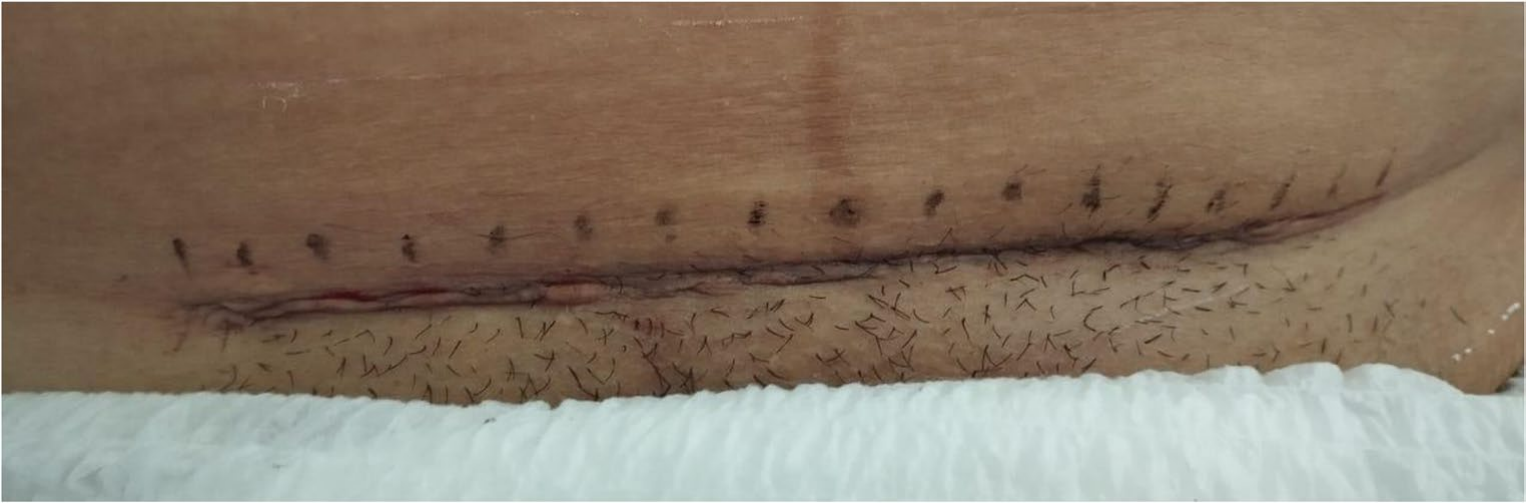
Laser therapy target points with a distance of 1 cm between them

Figure 1. Target points for laser therapy spaced 1 cm apart, determined according to incision length.

Laser application was performed directly over the cesarean incision, with participants positioned in the supine position and lower limbs flexed. Both participants and researchers wore protective eyewear during the procedure. The laser probe was positioned perpendicular to the skin along the incision line to minimize energy loss. The number of application points was determined according to the length of the surgical wound, with 1-cm spacing between points, measured using a sterile measuring tape (Fig. 1). The control group received standard postoperative care, including removal of the surgical dressing after the first 12 h postpartum and routine wound hygiene with soap and water during bathing. The laser equipment was disinfected with 70% alcohol after each procedure.

**Fig. 2:**
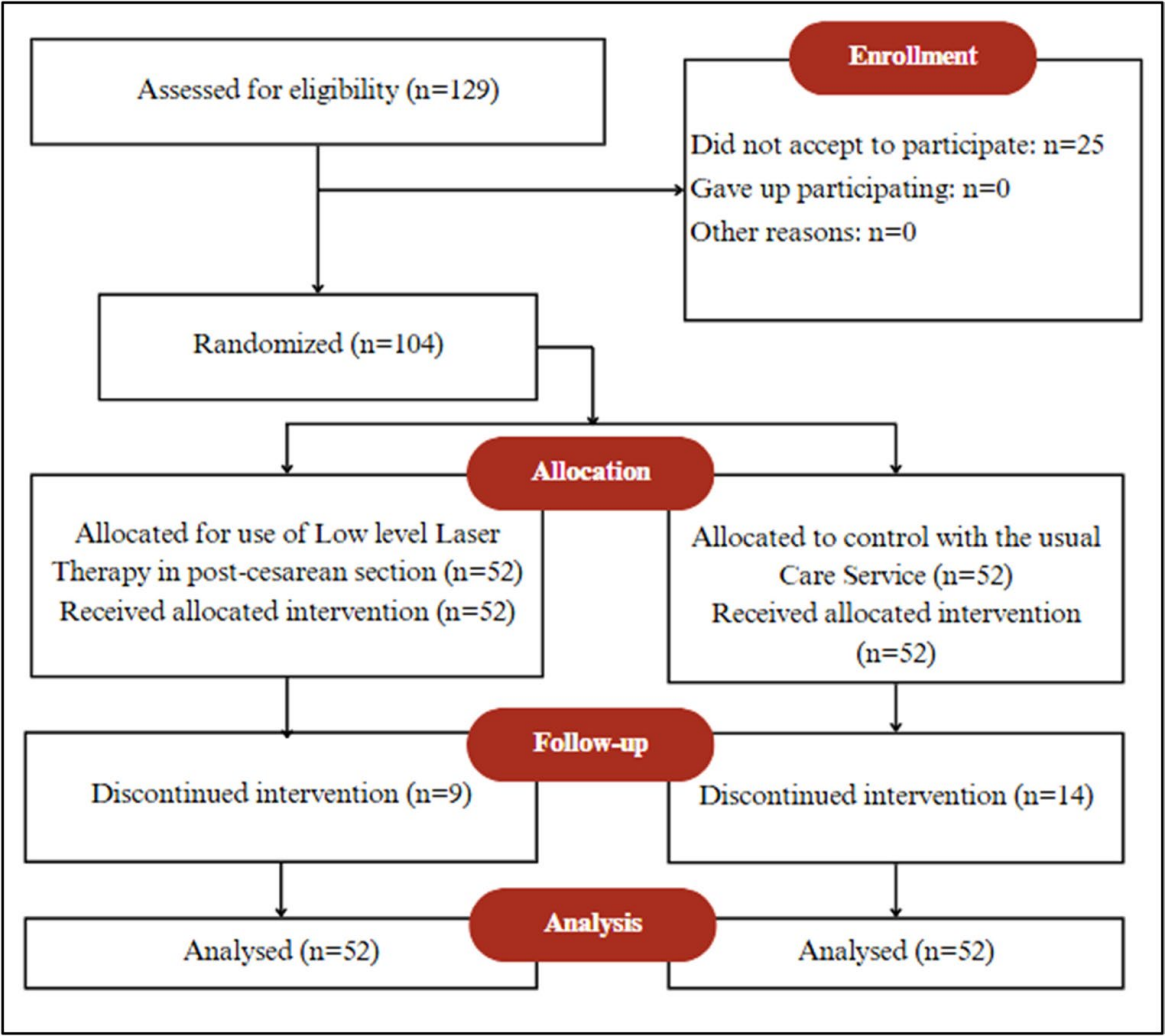
Participant flow diagram. Flowchart of participants

LLLT was applied in two phases: the first session was performed 24–30 h after cesarean section, and the second session occurred 24 h after the first assessment, corresponding to 48 h postoperatively (Fig. 2).

Pain intensity and wound healing quality were defined as the primary outcomes. Pain intensity was assessed using the Visual Analog Scale (VAS), which ranges from 0 (no pain) to 10 (worst pain imaginable) and was self-reported by the participants [8]. Wound healing quality was evaluated using the Vancouver Scar Scale (VSS), which assesses pigmentation (0–2), vascularity (0–3), pliability (0–5), and thickness (0–3), yielding a total score ranging from 0 to 13, with lower scores indicating better scar quality [9].

Secondary outcomes included the Global Perceived Change in Health Status, measured using a 19-item scale that assesses perceived changes in health status with three Likert-type response options: worse than before, no change, or better than before. This instrument comprises three domains: (a) occupation and physical health; (b) psychological aspects and sleep; and (c) relationships and emotional stability [10]. The need for pharmacological analgesia was also assessed based on medical records, defined as the request for additional analgesic doses. Adverse events, including blistering, redness, itching, and edema, were monitored during the intervention period.

Statistical analyses were conducted using R software version 4.2.2 for Mac OS X. Sociodemographic and clinical characteristics were summarized using measures of central tendency (means), dispersion (standard deviation or minimum and maximum values), and absolute and relative frequencies for categorical variables. Missing data were handled using multiple imputation with the Predictive Mean Matching (PMM) method via the *mice* package. Five imputed datasets were generated and pooled to ensure robustness of the analyses, with imputation applied to all variables with missing data while preserving data structure and variable relationships.

The effects of the intervention on study outcomes were analyzed according to the intention-to-treat principle. Linear mixed-effects models with restricted maximum likelihood estimation were used to estimate marginal mean differences between treatment groups (experimental vs. control) for the outcomes of interest at the end of the intervention period. These models included random intercepts and fixed effects for time, treatment, and their interaction (treatment × time). Differences in the proportions of global perceived change, adverse events, and need for analgesia between groups were assessed using Fisher’s exact test. Relative risk was calculated for the outcome of pharmacological analgesia use. The worst-case scenario was assumed for missing data. Statistical significance was set at *p* < 0.05. 

## Results

### Participant flow

A total of 129 postpartum women were assessed for eligibility, of whom 25 declined to participate. Consequently, 104 participants were randomized and allocated equally to the two study groups (*n* = 52 per group). Of the 104 randomized participants, 23 did not complete the second assessment scheduled at 48 h and were considered lost to follow-up. The primary reason for the absence of the second assessment was early hospital discharge, in accordance with routine maternity care. Importantly, these participants were retained in the analysis through multiple imputation and an intention-to-treat approach, thereby minimizing potential bias related to loss to follow-up. The reason for discontinuation at the second assessment was therefore early hospital discharge, consistent with institutional routine (Fig. 2).

### Participant characteristics

The groups were similar in terms of their baseline characteristics at the time of study inclusion (Table 1).

**Table 1:** Baseline characteristics of participants according to group allocation

Variables	Control (*n* = 52)	Experimental (*n* = 52)
Age, years [Min-Max]	30 ± 6 [19–43]	30 ± 6 [20–42]
Pregnancies, number [Min-Max]	2,4 ± 1,4 [1–6]	2,6 ± 1,9 [1–11]
Parity, number [Min-Max]	2,1 ± 1,2 [1–5]	2,1 ± 1,2 [1–5]
Gestational age, months [Min-Max]	37,9 ± 1,8 [31–41]	37,9 ± 1,4 [34–41]
Body mass, kg	77,6 ± 17,3	87,5 ± 18,5
Height, cm	160 ± 6,6	160 ± 6,2
Body mass index, kg/m2	29,9 ± 5,6	34,4 ± 6,7
History of abortion, n (% yes)	16 (28,8)	16 (30,7)
*Marital status*,* n (%)*		
Married/Stable union	24 (46,1)	22 (42,3)
Single/Divorced	28 (53,8)	30 (57,2)
*Education*,* n (%)*		
1–3 years	1 (2,0)	3 (5,8)
4–7 years	15 (28,8)	15 (28,8)
8–11 years	28 (53,8)	28 (53,8)
≥ 12 years	8 (15,4)	6 (11,6)
*Income*,* n (%)*		
< 1 minimum wage	30 (39,2)	27 (51,9)
≥ 1 minimum wage	31 (60,8)	25 (48,1)
*Origin*,* n (%)*		
Interior PE	25 (49,0)	20 (38,5)
MRR	26 (51,0)	32 (61.5)

*RMR* Metropolitan Region of Recife, *Min* Minimum, *Max* Maximum, *PE* Pernambuco

Minimum salary in Brazil 2024: R$1,412

## Participant characteristics

The groups were comparable with respect to baseline characteristics at the time of study inclusion (Table 1).

**Table 2:** Group means and mean differences (95% CI) for VAS pain and Vancouver Scar Scale at 48-hour follow-up

Desfecho	Controle*n* = 52	Experimental*n* = 52	DM (95%CI)Experimental vs. Control
	Reavaluation − 48 h(Mean ± EPE)	Reavaluation − 48 h(Mean ± EPE)	Reavaluation − 48 h
EVA (0–10)	5.5 ± 0,3	4.1 ± 0.3	-1.4 (-2.3; -0.5)
Vancouver (0–13)	5,07 ± 0,32	1,76 ± 0,30	3,31 (3.19; 3.43)
Thickness (0–3)	1,39 ± 0,13	1,11 ± 0,12	-0,28 (-0.63; 0.07)
Vascularization (0–3)	1,41 ± 0,07	0,41 ± 0,08	1.0 (1.00; 1.03)
Pigmentation (0–2)	0,12 ± 0,06	0,17 ± 0,06	0,08 (-0,11; 0,27)
Flexibility (0–5)	1,18 ± 0,11	0,07 ± 0,12	1.11 (1.07; 1.15)

VAS− Visual Analogue Scale (0 – 10). Low values indicate reduced pain perception. Vancouver Scar Scale (0 – 13). Low values indicate better quality of healing. MD− Estimated mean difference; EPE− Standard error of estimate

### Effects of the intervention

#### Primary outcomes

A significant reduction in pain intensity was observed at 48 h in the LLLT group compared with standard care, favoring the intervention group (mean difference [MD]: −1.4; 95% CI: −2.3 to − 0.5) (Table 2).

Regarding wound healing quality, LLLT demonstrated a significant difference compared with standard care based on the total Vancouver Scar Scale score at 48 h, with an MD of 3.31 (95% CI: 3.19 to 3.43) (Table 2).

Domain-specific analysis of the Vancouver Scar Scale revealed significant differences only in vascularity at 48 h (MD: 1.0; 95% CI: 1.00 to 1.03) and scar pliability at 48 h (MD: 1.11; 95% CI: 1.07 to 1.15). No significant differences were observed in the thickness or pigmentation domains (Table 2).

#### Secondary outcomes

No significant differences were observed between groups in global perceived health status at 48 h (*p* = 0.848) or in the need for pharmacological analgesia at 48 h (relative risk [RR]: 1.70; 95% CI: 0.86 to 3.36) (Table 3).

**Table 3:** Proportions of global health change perception and requests for pharmacologic analgesia by group

Outcomes	Reavaluation − 48 h			
	No change	Worse than before	Better than before	*P*
Global health perception	*n*(%)	*n*(%)	*n*(%)	
Control (*n* = 52)	4 (3,8)	1 (2)	46 (44,2)	0,848
Experimental (*n* = 52)	12 (11,5)	1 (1)	39 (37,5)	
	*No*	*Yes*	*RR (IC95%)*	*P*
Pharmacological analgesia				0.434
Control (*n* = 52)	42 (40,4)	10 (9,6)	1.70 (0,86;3,36)	
Experimental (*n* = 52)	35 (33,6)	17 (16,4)		

*RR* Relative Risk, *CI* Confidence Interval

## Discussion

This study demonstrated improvements in pain intensity and surgical wound healing quality in postpartum women undergoing cesarean section treated with low-level laser therapy (LLLT) compared with standard postoperative care. However, no significant effects were observed regarding the need for pharmacological analgesia or global perceived health status.

Although the observed reduction in pain intensity was statistically significant, its clinical relevance should also be considered. A mean difference of 1.4 points on the Visual Analog Scale may represent a meaningful improvement in patient comfort, particularly in the immediate postoperative period, when pain can directly interfere with mobility, maternal–infant interaction, and the ability to perform self-care activities. Therefore, even modest reductions in pain intensity may have important implications for functional recovery and overall postpartum well-being.

The reduction in pain intensity observed with LLLT applied to the cesarean surgical wound may be explained by physiological mechanisms related to the absorption of infrared light at a dose of 5 J/cm², which enhances adenosine triphosphate (ATP) production and accelerates cellular processes involved in tissue repair. These effects contribute to a reduction in local inflammation and a decrease in the release of chemical mediators such as prostaglandins, which are known to exacerbate postoperative pain [11].

In addition, LLLT stimulates the release of endorphins—neurotransmitters that function as endogenous analgesics—thereby contributing to pain relief [12]. LLLT is also capable of modulating nerve fibers involved in pain transmission by inhibiting the activity of C fibers, which are responsible for intensifying pain perception [13]. Another important effect of LLLT is the improvement of local blood flow in the treated area, facilitating the removal of inflammatory mediators and enhancing tissue oxygenation. The combination of these mechanisms supports the effectiveness of LLLT in pain control, particularly in postoperative pain conditions such as cesarean delivery [14].

Other studies investigating the use of LLLT in the postoperative period following cesarean section, using doses different from those applied in the present study, have also reported favorable outcomes regarding pain reduction [15]. One study evaluated the combined use of red and infrared light in the immediate postoperative period after cesarean delivery. The intervention was initiated in the operating room immediately after surgery and before wound dressing. A dose of 3 J/cm² (1 J red + 2 J infrared) was applied directly to the incision, and 6 J/cm² (4.5 J infrared + 1.5 J red) was applied to the surrounding tissue, totaling 27 J of energy per session [16]. The results showed accelerated wound healing in the laser-treated group, with a significant reduction in inflammatory signs according to the REEDA scale on postoperative days 3, 7, and 10. In addition, these patients reported lower pain intensity and greater aesthetic satisfaction with the scar compared with the control group, reinforcing the potential of low-level laser therapy as an effective adjunct for enhancing wound healing and postoperative comfort following cesarean section.

Another study investigated the effects of low-level laser therapy (LLLT) on post-cesarean pain. Participants were allocated into four groups: control, placebo, experimental group I (4 J/cm²), and experimental group II (2 J/cm²). Pain assessments using the Numeric Pain Rating Scale, pressure algometry, and the Global Perceived Effect Scale were performed at 12, 20–24, and 44–48 h after surgery. The results demonstrated a significant interaction between time and group for both pain scores and algometry measures. The Global Perceived Change Scale revealed significant differences between groups at 20–24 h (*p* = 0.04) and 44–48 h (*p* = 0.04). Both laser doses were effective in reducing post-cesarean pain, with no clinically significant differences between the doses [7].

Considering that both laser doses tested produced positive results in pain reduction without clinically meaningful differences between them, prioritizing the use of an effective dose appears reasonable. This approach optimizes application time, reduces operational costs, and facilitates the incorporation of the protocol into routine healthcare services. Furthermore, the use of a standardized single dose enhances safety, minimizes the risk of adverse effects, and improves adherence among both patients and healthcare professionals.

From a clinical perspective, the findings of this study support the use of LLLT as a feasible and low-cost adjunctive therapy in post-cesarean care, particularly in public healthcare settings. The intervention is non-invasive, easy to apply, and can be integrated into routine clinical practice without significant additional burden to healthcare professionals. Even in the absence of reductions in analgesic consumption, improvements in pain intensity and wound healing may contribute to enhanced patient comfort, earlier mobilization, and potentially better postpartum recovery trajectories.

Considering that both laser doses tested produced positive results in pain reduction without clinically meaningful differences between them, prioritizing the use of an effective dose appears reasonable. This approach optimizes application time, reduces operational costs, and facilitates the incorporation of the protocol into routine healthcare services. Furthermore, the use of a standardized single dose enhances safety, minimizes the risk of adverse effects, and improves adherence among both patients and healthcare professionals.

From a clinical perspective, the findings of this study support the use of LLLT as a feasible and low-cost adjunctive therapy in post-cesarean care, particularly in public healthcare settings. The intervention is non-invasive, easy to apply, and can be integrated into routine clinical practice without significant additional burden to healthcare professionals. Even in the absence of reductions in analgesic consumption, improvements in pain intensity and wound healing may contribute to enhanced patient comfort, earlier mobilization, and potentially better postpartum recovery trajectories.

Corroborating these findings, another study evaluated the safety of LLLT in the post-cesarean period, particularly concerning prolactin levels and surgical incision healing. In this randomized clinical trial, a gallium–aluminum–arsenide (GaAlAs) diode laser was used at two different dosages: 4.8 J/cm² (3.3 J/cm² infrared and 1.5 J/cm² red) and 2.9 J per point (2 J infrared and 0.9 J red), applied over three consecutive sessions beginning immediately after cesarean delivery. The control group received only conventional postoperative care. The results showed no significant differences in prolactin levels between groups, indicating that LLLT does not interfere with lactation. Conversely, laser-treated areas exhibited reduced inflammation and increased vascularization, suggesting beneficial effects on wound healing [17].

The physiological mechanism underlying the effects of LLLT on postoperative wound healing is believed to involve absorption by cytochrome c oxidase (CCO), which enhances mitochondrial activity and cellular metabolism, promoting tissue repair [18, 19]. These processes result in increased ATP synthesis, modulation of inflammation, and stimulation of cellular proliferation, all of which contribute to improved healing outcomes.

Regarding wound healing assessment, superior outcomes were observed in the LLLT group in terms of vascularity and pliability—two essential components for optimal scar formation. Adequate vascularization ensures efficient delivery of oxygen and nutrients, while improved tissue flexibility contributes to better functional recovery and reduced risk of restrictive scar formation [20].

Although differences in vascularity and pliability parameters were observed between groups, it is important to emphasize that the assessment was conducted in the early postoperative period (48 h), which does not allow inferences regarding definitive scar formation and maturation. Therefore, these findings should be interpreted as early indicators of tissue repair rather than evidence of established improvements in wound healing. This limitation was considered in the interpretation of the results and highlights the need for studies with medium- and long-term follow-up. The results suggest a potential positive effect of the intervention on early aspects of the tissue repair process; however, these findings should be interpreted with caution, given the short evaluation period.

No adverse effects associated with LLLT were observed in this study. Adverse events were actively monitored during and after each intervention session through clinical observation and patient self-report, including signs such as hyperemia, edema, pruritus, and blister formation. This finding is consistent with previous research and reinforces the safety profile of LLLT in post-cesarean care.

Although it was anticipated that women receiving LLLT would report improvements in perceived health status and reduced need for pharmacological analgesia, no significant differences were observed between groups. This finding may be explained by the short follow-up period and the standardized use of analgesic protocols in the immediate postoperative setting, which may have reduced the sensitivity of these outcomes to detect between-group differences. Additionally, global health perception is a multidimensional construct that may not change substantially within the first 48 h after surgery. These findings suggest that longer follow-up periods may be necessary to fully capture the broader clinical effects of LLLT.

Corroborating these findings, another study evaluated the safety of LLLT in the post-cesarean period, particularly concerning prolactin levels and surgical incision healing. In this randomized clinical trial, a gallium–aluminum–arsenide (GaAlAs) diode laser was used at two different dosages: 4.8 J/cm² (3.3 J/cm² infrared and 1.5 J/cm² red) and 2.9 J per point (2 J infrared and 0.9 J red), applied over three consecutive sessions beginning immediately after cesarean delivery. The control group received only conventional postoperative care. The results showed no significant differences in prolactin levels between groups, indicating that LLLT does not interfere with lactation. Conversely, laser-treated areas exhibited reduced inflammation and increased vascularization, suggesting beneficial effects on wound healing [17].

The physiological mechanism underlying the effects of LLLT on postoperative wound healing is believed to involve absorption by cytochrome c oxidase (CCO), which enhances mitochondrial activity and cellular metabolism, promoting tissue repair [18, 19]. These processes result in increased ATP synthesis, modulation of inflammation, and stimulation of cellular proliferation, all of which contribute to improved healing outcomes.

Regarding wound healing assessment, superior outcomes were observed in the LLLT group in terms of vascularity and pliability—two essential components for optimal scar formation. Adequate vascularization ensures efficient delivery of oxygen and nutrients, while improved tissue flexibility contributes to better functional recovery and reduced risk of restrictive scar formation [20].

Although differences in vascularity and pliability parameters were observed between groups, it is important to emphasize that the assessment was conducted in the early postoperative period (48 h), which does not allow inferences regarding definitive scar formation and maturation. Therefore, these findings should be interpreted as early indicators of tissue repair rather than evidence of established improvements in wound healing. This limitation was considered in the interpretation of the results and highlights the need for studies with medium- and long-term follow-up. The results suggest a potential positive effect of the intervention on early aspects of the tissue repair process; however, these findings should be interpreted with caution, given the short evaluation period.

No adverse effects associated with LLLT were observed in this study. Adverse events were actively monitored during and after each intervention session through clinical observation and patient self-report, including signs such as hyperemia, edema, pruritus, and blister formation. This finding is consistent with previous research and reinforces the safety profile of LLLT in post-cesarean care.

Although it was anticipated that women receiving LLLT would report improvements in perceived health status and reduced need for pharmacological analgesia, no significant differences were observed between groups. This finding may be explained by the short follow-up period and the standardized use of analgesic protocols in the immediate postoperative setting, which may have reduced the sensitivity of these outcomes to detect between-group differences. Additionally, global health perception is a multidimensional construct that may not change substantially within the first 48 h after surgery. These findings suggest that longer follow-up periods may be necessary to fully capture the broader clinical effects of LLLT.

This study is pioneering in evaluating the use of infrared light at a dose of 5 J/cm² for both pain reduction and wound healing quality in postpartum women following cesarean section. By simultaneously addressing these clinically relevant outcomes, the study contributes to a more comprehensive understanding of post-cesarean recovery.

Some limitations of this study should be acknowledged. Sample attrition occurred due to early hospital discharge, which may have influenced follow-up completeness; however, intention-to-treat analysis was used to mitigate potential bias. Pain assessment was limited to a unidimensional measure (VAS), which does not capture emotional, functional, and psychosocial dimensions. The short follow-up period restricted the evaluation of long-term outcomes such as persistent pain and scar maturation. Additionally, clinically relevant outcomes such as patient satisfaction, aesthetic perception, functional recovery, and return to daily activities were not assessed. Finally, as this was a single-center study, caution should be exercised when generalizing the findings to other populations and healthcare settings. Nevertheless, the study was conducted in a real-world public hospital setting with a socioeconomically diverse population, which may enhance its external validity within similar contexts.

## Conclusion

The findings of the present study indicate that low-level laser therapy (LLLT) is effective in reducing pain intensity and improving surgical wound healing in the immediate postoperative period following cesarean section, with more pronounced effects observed in the domains of scar vascularity and pliability. However, LLLT did not demonstrate a significant impact on global perceived health status nor did it result in a reduction in the need for pharmacological analgesia during the follow-up period. Given the relatively short duration of follow-up, future studies with larger sample sizes and medium- to long-term follow-up are recommended to confirm these findings and to further elucidate the role of LLLT in post-cesarean recovery protocols. 

## Data Availability

No datasets were generated or analysed during the current study.
